# Ferric Iron Reduction in Extreme Acidophiles

**DOI:** 10.3389/fmicb.2021.818414

**Published:** 2022-01-12

**Authors:** Luise Malik, Sabrina Hedrich

**Affiliations:** Research Group Biohydrometallurgy and Microbiology, Institute of Biosciences, TU Bergakademie Freiberg, Freiberg, Germany

**Keywords:** extreme acidophiles, iron reduction, biochemistry, biohydrometallurgy, reductive bioleaching

## Abstract

Biochemical processes are a key element of natural cycles occurring in the environment and enabling life on earth. With regard to microbially catalyzed iron transformation, research predominantly has focused on iron oxidation in acidophiles, whereas iron reduction played a minor role. Microbial conversion of ferric to ferrous iron has however become more relevant in recent years. While there are several reviews on neutrophilic iron reducers, this article summarizes the research on extreme acidophilic iron reducers. After the first reports of dissimilatory iron reduction by acidophilic, chemolithoautotrophic *Acidithiobacillus* strains and heterotrophic *Acidiphilium* species, many other prokaryotes were shown to reduce iron as part of their metabolism. Still, little is known about the exact mechanisms of iron reduction in extreme acidophiles. Initially, hypotheses and postulations for the occurring mechanisms relied on observations of growth behavior or predictions based on the genome. By comparing genomes of well-studied neutrophilic with acidophilic iron reducers (e.g., *Ferroglobus placidus* and *Sulfolobus* spp.), it became clear that the electron transport for iron reduction proceeds differently in acidophiles. Moreover, transcriptomic investigations indicated an enzymatically-mediated process in *Acidithiobacillus ferrooxidans* using respiratory chain components of the iron oxidation in reverse. Depending on the strain of *At. ferrooxidans*, further mechanisms were postulated, e.g., indirect iron reduction by hydrogen sulfide, which may form by disproportionation of elemental sulfur. Alternative scenarios include Hip, a high potential iron-sulfur protein, and further cytochromes. Apart from the anaerobic iron reduction mechanisms, sulfur-oxidizing acidithiobacilli have been shown to mediate iron reduction at low pH (< 1.3) under aerobic conditions. This presumably non-enzymatic process may be attributed to intermediates formed during sulfur/tetrathionate and/or hydrogen oxidation and has already been successfully applied for the reductive bioleaching of laterites. The aim of this review is to provide an up-to-date overview on ferric iron reduction by acidophiles. The importance of this process in anaerobic habitats will be demonstrated as well as its potential for application.

## Lifestyle of Acidophilic Microorganisms

Acidic environments such as acid mine drainage are the result of anthropogenic influences or have a natural origin, e.g., solfataric springs (Johnson, 2009). Depending on the occurring pH, these environments are habitats for moderate (pH 3–5) or extreme acidophilic (pH < 3) organisms. Representatives of all three domains have been identified under acidic conditions, also eukaryotes, mainly fungi and algae, some rotifers, ciliates and species of the genus *Euglena*, were determined in acidic waterbodies (Quatrini and Johnson, 2016; Stolz, 2017; Johnson and Quatrini, 2020). However, this article will focus on acidophilic prokaryotes, which are well adapted to the challenging conditions of extremely low pH habitats (Dopson, 2016; Golyshina et al., 2016).

### Adaptation to Extreme Environments

Most of the challenges acidophiles have to face with respect to their metabolism and morphological adaptations, are dictated by the conditions in their extreme environment. Low pH values are characterized by high concentrations of hydronium ions caused by a combination of proton-producing biotic and abiotic reactions, for instance, the generation of sulfuric acid in mine-impacted and geothermal areas (Johnson, 2009). Acidophiles have evolved appropriate mechanisms to overcome this cell homeostasis-threatening issue, e.g., the presence of a reverse positive inner membrane potential (Matin, 1999). With respect to highly acidic habitats, high temperatures are also an important influence, as these two quite hostile conditions occasionally appear together, for example, in hot solfatara springs or volcanoes (Johnson, 2009). Besides high concentrations of protons, a variety of metal cations is present in most acidic environments. These are generated by mineral leaching processes, which cause mobilization of partially toxic metal and metalloid cations (Gadd, 2009). The cations present, especially iron and copper, can in turn generate reactive oxygen species, e.g., through the Fenton reaction, which increases the oxidative stress and causes damage of cellular components (Ferrer et al., 2016). However, the inorganic compounds released during leaching processes are also a source of energy for many microorganisms. This is of particular importance since most acidic environments have low levels of organic carbon sources which forced the development of widespread abilities to utilize autotrophic metabolisms (Johnson, 2009).

### Metabolic Diversity of Extreme Acidophiles

**FIGURE 1:**
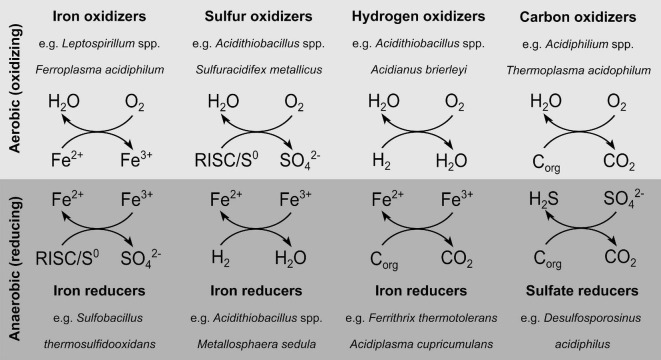
Diversity of dissimilation pathways used by acidophiles. The underlying data can be found in the associated studies (Hedrich et al., 2011; Hedrich and Johnson, 2013b; Dopson, 2016). RISC, reduced inorganic sulfur compounds; C_org_, organic carbon.

Despite the fact that acidophiles are to some extent constrained by their environmental conditions, diverse metabolisms have evolved that are used by these organisms (Figure 1; Johnson, 1998). Acidophiles can exhibit both obligate heterotrophic or obligate/facultative autotrophic carbon metabolisms, with the latter using oxidation or reduction of elemental sulfur, iron or hydrogen to generate ATP (Johnson and Hallberg, 2008; Johnson, 2009). Some acidophiles use a wide range of dissimilation pathways, while the obligate aerobic autotrophic *Leptospirillum* is only capable of oxidizing ferrous iron (Dopson, 2016). Depending on the utilized electron acceptor and donor, the acidophilic electron transport systems differ considerably. Especially in the case of sulfur metabolism, the variety of occurring oxidation states (–2 to + 6) allow several pathways by which reduced inorganic sulfur compounds (RISC) are partially or completely oxidized to sulfate. Nevertheless, a lot of effort was put in the elucidation of the underlying enzymatic processes, as reviewed elsewhere (Wang et al., 2019; Dahl, 2020). In addition to the well-studied sulfur metabolism, hydrogen oxidation under anaerobic or aerobic conditions is also a common feature of acidophiles (Hedrich and Johnson, 2013b; Kucera et al., 2020).

**FIGURE 2:**
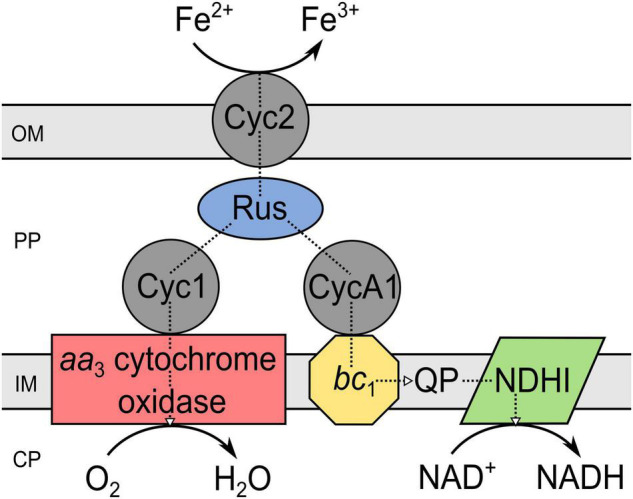
Components of the iron oxidation pathway in *Acidithiobacillus ferrooxidans*. The scheme is a modified version of that given by Ilbert and Bonnefoy (2013). PP, periplasm; CP, cytoplasm; OM, outer membrane; IM, inner membrane; Cyc, cytochrome *c*; Rus, rusticyanin; QP, quinol pool; NDHI, NADH dehydrogenase complex; *bc*_1_, cytochrome *bc*_1_ complex (encoded by *petI/II*).

Regarding the iron oxidation mechanism in acidophiles, different strategies evolved simultaneously in several phyla, e.g., γ-*Proteobacteria*, *Firmicutes*, *Nitrospira*, *Crenarchaeota*, and *Euryarchaeota* (Ilbert and Bonnefoy, 2013). In particular, for *Acidithiobacillus ferrooxidans*, bioinformatic, genetic, proteomic, and transcriptomic investigations of the electron transfer chain and related genes led to a widely accepted iron oxidation model (Ingledew, 1982; Bonnefoy, 2010; Amouric et al., 2011; Kucera et al., 2013; Jiang et al., 2021). This is still work in progress, as the results obtained on an ongoing basis extend the previously postulated iron oxidation mechanism to some extent, for example through newly discovered gene clusters (Ai et al., 2018). Nevertheless, the acknowledged electron transport chain from iron to the terminal electron acceptor includes an outer membrane cytochrome *c* (Cyc2), transferring the electron from iron to rusticyanin, a copper protein situated in the periplasm (Giudici-Orticoni et al., 1999; Yarzábal et al., 2002). Depending on its destination, the electron is further transported to the NADH-1 complex *via* cytochrome *c* (CycA1), the cytochrome *bc*_1_ complex and ubiquinone pool to produce reduction equivalents (Elbehti et al., 1999, 2000). Electron transport along the thermodynamic gradient to reduce oxygen to water occurs, if the electron is transferred from rusticyanin to cytochrome *c* (Cyc1) and further to the *aa*_3_ cytochrome oxidase (Kai et al., 1992; Malarte et al., 2005). Accordingly, the components of the iron oxidation chain in *Acidithiobacillus ferrooxidans* form a super-complex extending from the outer membrane integrated Cyc2 through the periplasm to the cytochrome oxidase, *bc*_1_ complex, and NADH-1 complex in the inner membrane (Figure 2; Ilbert and Bonnefoy, 2013).

Many acidophiles are facultative anaerobes, preferentially using oxygen as electron acceptor, but in its absence, they may alternatively reduce iron or rarely sulfur (Gyure et al., 1990; Coupland and Johnson, 2008). Dissimilatory ferric iron reduction under anaerobic conditions is widespread among acidophiles and can be coupled to the oxidation of hydrogen, sulfur, RISC or organic compounds (Coupland and Johnson, 2008; Johnson, 2010). The greater iron solubility at low pH and its abundance in many acidic environments are reasons why iron is used by acidophiles as both electron acceptor and donor (Johnson, 2009). Additionally, the much higher redox potential of the ferrous/ferric iron pair at low pH (+ 663 mV, pH 2, sulfate-rich solution) (Johnson et al., 2017) makes it a more feasible electron acceptor compared to neutral conditions (+ 200 mV, pH 7, bicarbonate-containing environments) (Ehrenreich and Widdel, 1994). Accordingly, microbial cycling of iron can occur between habitat niches of different dissolved oxygen levels, which contributes significantly to the natural iron cycle.

## Iron Reduction by Acidophiles

Iron reduction as part of the natural iron cycle plays an important role on Earth (Kappler and Straub, 2005). Despite the well-studied mechanisms of iron reduction by neutrophilic microorganisms, the process in acidophilic microbes has not yet been sufficiently explored. Nevertheless, genome comparisons of known neutrophilic iron reducers with those of acidophiles revealed few similarities suggesting a distinct mechanism in acidophiles (Masaki et al., 2018). Depending on the considered microorganism, ferric iron reduction by acidophiles can take place under anaerobic, aerobic as well as micro-aerobic conditions. The most important milestones of ferric iron reduction research in acidophiles are summarized in Table 1. First investigations of acidithiobacilli described aerobic ferric iron reduction at low pH (Brock and Gustafson, 1976; Sand, 1989) and anaerobic ferrous iron formation (Brock and Gustafson, 1976). Micro-aerobic iron reduction was especially studied for the genus *Acidiphilium* (Johnson and Bridge, 2002). Regarding archaea, only a few selected genera have been studied profoundly with respect to their iron reduction mechanism, most notably the anaerobic and micro-aerobic studies of *Sulfolobus*, *Sulfuracidifex*, and *Saccharolobus* (latter no longer termed as extreme acidophile due to a pH optimum of 4.5) (Masaki et al., 2018). Moreover, investigations of the archeon “*Ferroplasma acidarmanus*” Fer1 revealed anaerobic ferric iron reduction rates which were comparable with those determined for *Acidiphilium* species (Dopson et al., 2007). Overall, ferric iron reduction was shown for different physiologic groups of acidophilic microorganisms, covering heterotrophs and chemoautotrophs which feature temperature optima ranging from mesophilic to thermophilic adaptations (Johnson and Bridge, 2002). In particular, the ability of acidophiles to mediate reductive dissolution of ferric iron minerals is frequently explored, with emphasis on the applicability prior to elucidation of the exact biochemical mechanism (Hallberg et al., 2011a; Nancucheo et al., 2014; Norris et al., 2015; Marrero et al., 2017). However, certain research groups provided insight into the mechanisms of ferric iron reduction that occur in extreme acidophiles at different oxygen concentrations, which will be discussed in more detail.

**TABLE 1:** Most important discoveries during research of iron reduction in acidophiles.

Year	Investigation	References
1976	First description of ferric iron reduction by acidophilic bacteria and archaea: • *Acidithiobacillus thiooxidans* (aerobic) • *Acidithiobacillus ferrooxidans* (anaerobic) • *Sulfolobus acidocaldarius* (aerobic)	Brock and Gustafson, 1976
1987	Purification and characterization of a sulfur:ferric iron oxidoreductase from ferrous iron-grown *At. ferrooxidans*	Sugio et al., 1987
1987	First model of anaerobic iron respiration pathway of *At. ferrooxidans* utilizing transporters of the ferrous iron oxidation chain (cytochrome *bc*_1_ complex, cytochrome *c*, rusticyanin, cytochrome oxidase, cytochrome *a*_1_)	Corbett and Ingledew, 1987
1989	Aerobic iron reduction by resting cells of *At. ferrooxidans* at extremely low pH	Sand, 1989
1991	Anaerobic dissimilatory iron reduction by *At. ferrooxidans*	Pronk et al., 1991
1991	Aerobic dissimilatory iron reduction by heterotrophic *Acidiphilium* spp.	Johnson and McGinness, 1991
1998	Iron reduction by moderately thermophilic iron-oxidizing bacteria: • *Sulfobacillus thermosulfidooxidans* • *Sulfobacillus acidophilus* • *Acidimicrobium ferrooxidans*	Bridge and Johnson, 1998
	Iron cycling in static, aerobic cultures	
2002	Distinction between a constitutive and oxygen-inducible iron reduction system in *Acidiphilium* spp.	Johnson and Bridge, 2002
2011	First demonstration of using iron reduction by acidophiles for mineral processing and metal extraction	Hallberg et al., 2011a
2013	Suggestion of a second, indirect iron reduction mechanism in *At. ferrooxidans* mediated by H_2_S at low pH	Osorio et al., 2013
2015	Indication of an indirect, non-growth-related iron reduction mechanism in *At. thiooxidans* at aerobic, extreme acidic conditions	Marrero et al., 2015
2016	Hypothetic iron reduction pathway in *At. ferrooxidans* including a high potential iron-sulfur protein	Kucera et al., 2016a
2020	Model for anaerobic iron reduction coupled to hydrogen oxidation in *At. ferrooxidans*	Kucera et al., 2020
2021	Introduction of the term “latent iron reduction” for acidithiobacilli	Johnson et al., 2021

### Anaerobic Iron Reduction at Low pH

After the observation of iron-reducing processes by numerous neutrophilic prokaryotes (Bromfield, 1954; Ottow and Glathe, 1971; Lovley, 1991), anaerobic iron reduction by extreme acidophiles was first described in 1976, for the species *Acidithiobacillus ferrooxidans* (Brock and Gustafson, 1976). Under the given conditions, elemental sulfur-respiring *At. ferrooxidans* reduced iron exclusively in the absence of oxygen. Accordingly, it was hypothesized that ferric iron may serve as an alternative electron acceptor for acidophiles in anaerobic habitats. To date, iron reduction in the absence of oxygen has been demonstrated for a variety of moderate and extremely acidophilic microorganisms. The phylogenetically heterogenous group of iron-reducing acidophiles includes archaeal as well as bacterial species covering the phyla *Crenarchaeota*, *Euryarchaeota*, *Acidobacteria*, *Actinobacteria*, *Firmicutes*, and *Proteobacteria* (Tables 2–5).

**TABLE 2:** Ferric iron-reducing extreme acidophilic archaea.

Organism	Fe ox.	S^0^ ox.	pH	Temp. [°C]	Conditions Fe red., ED	Growth	References
*Acidianus brierleyi*^T^**	+	+	1.5–2.0 1.0–6.0	70 45–75	Anaerobic: H_2_S (w/, w/o OC)	+	Plumb et al., 2007
*“Acidianus copahuensis”*	+	+	2.5–3.0 1.0–5.0	75 55–80	Anaerobic: S^0^ or H_2_	+	Giaveno et al., 2013
*“Acidianus manzaensis”* ^T^	–	+	1.2–1.5 1.0–5.0	80 60–90	Anaerobic: S^0^, H_2_ or OC	+	Yoshida et al., 2006
*Acidianus sulfidivorans* ^T^	+	+	0.8–1.4 0.35–3.0	74 45–83	Anaerobic: H_2_S (w/, w/o OC)	+	Plumb et al., 2007
*Acidiplasma aeolicum* ^T^	+	RISC	1.4–1.6 0–4.0	42–45 15–65	Anaerobic: OC	+	Golyshina et al., 2009
*Acidiplasma cupricumulans* ^T^	+	–	1.0–1.2 > 0.4	53.6 22–63	Anaerobic: K_2_S_4_O_6_ w/OC	+	Hawkes et al., 2006
*“Ferroplasma acidarmanus”* ^T^	+	ND	1.2 0.2–2.5	42 23–46	Anaerobic: OC	+	Dopson et al., 2004
*Ferroplasma acidiphilum*^T^, DR1 and MT17	+	–RISC	1.7 1.3–2.2	35 20–45	Anaerobic: OC	+	Dopson et al., 2004; Johnson, 2010
*“Ferroplasma thermophilum”*	+	–	1.0 0.2–2.5	45 30–60	Anaerobic: OC	+	Zhou et al., 2008
*Metallosphaera sedula* ^T^	+	+	1.0–4.5	75 50–80	Aerobic: H_2_	ND	Auernik and Kelly, 2010
*Saccharolobus caldissimus* ^T^	FeS_2_	–	3.0 1.5–6.0	85 65–93	Anaerobic: OC	+	Sakai and Kurosawa, 2018
*Saccharolobus shibatae* ^T^	FeS_2_	–	3.0 1.5–6.0	81 55–86	Anaerobic: OC	+	Sakai and Kurosawa, 2018
*Sulfolobus acidocaldarius* ^T^	+	+	2.0–3.0 1.0–5.9	75–80 55–80	Aerobic: S^0^ or OC Microaerobic: OC Anaerobic: OC	– ND –	Brock and Gustafson, 1976; Masaki et al., 2018
*Sulfuracidifex metallicus* ^T^	+	+	2.0–3.0 1.0–4.5	65 50–75	Anaerobic: S^0^	–	Masaki et al., 2018
*Sulfurisphaera ohwakuensis* ^T^	FeS_2_	+	2.0 1.5–6.0	84 60–91	Anaerobic: OC	+	Tsuboi et al., 2018
*Sulfurisphaera tokodaii* ^T^	+	+	2.5–3.0 1.5–6.0	80 60–96	Anaerobic: S^0^ or OC Micro-aerobic: OC	+ ND	Masaki et al., 2018
*Thermoplasma acidophilum* ^T^	ND	–	1.0–2.0 0.5–34.0	59 45–63	Anaerobic: S^0^	ND	Zhang et al., 2018

*pH, temperature, sulfur oxidation and iron oxidation data correspond to the optima and range, which are described in the respective characterization publications. The growth column indicates whether dissimilatory iron reduction occurs under the given conditions. Temp, temperature; ox, oxidation; red, reduction; ED, electron donor; ND, no data; OC, organic carbon.*

**TABLE 3:** Ferric iron-reducing extreme acidophilic autotrophic bacteria.

Organism	Fe ox.	S^0^ ox.	pH	Temp. [°C]	Conditions Fe red., ED	Growth	References
*Acidiferrobacter thiooxydans* ^T^	+	+	2.0 > 1.2	38 < 47	Anaerobic: S^0^	+	Hallberg et al., 2011b
*Acidithiobacillus caldus* ^T^	–	+	2.0–2.5 1.0–3.5	45 32–52	Aerobic: S^0^	–	Johnson et al., 2017
*Acidithiobacillus ferrianus* ^T^	+	+	2.0	30	Anaerobic: S^0^ or H_2_	+	Norris et al., 2020
*Acidithiobacillus ferridurans* ^T^	+	+	2.1 < 1.3	29	Anaerobic: S^0^, K_2_S_4_O_6_ or H_2_ Aerobic: S^0^	+ –	Hedrich and Johnson, 2013a; Johnson et al., 2017
*Acidithiobacillus ferriphilus* ^T^	+	+	2.0	30	Anaerobic: RISC	+	Falagán and Johnson, 2016
*Acidithiobacillus ferrivorans*^T^, Peru6	+	+	2.5 1.9–3.4	27–32 4–37	Anaerobic: S^0^ Aerobic: S^0^	+ –	Hallberg et al., 2010; Johnson et al., 2017
*Acidithiobacillus ferrooxidans* ^T^	+	+	2.5 1.3–4.5	30–35 10–37	Anaerobic: S^0^ Aerobic: S^0^	– +	Pronk et al., 1992; Johnson et al., 2017
*Acidithiobacillus thiooxidans* ^T^	–	+	2.0–3.0 0.5–5.5	28–30 10–37	Aerobic: S^0^	–	Johnson et al., 2017

*pH, temperature, sulfur oxidation and iron oxidation data correspond to the optima and range, which are described in the respective characterization publications. The growth column indicates whether dissimilatory iron reduction occurs under the given conditions. Temp, temperature; ox, oxidation; red, reduction; ED, electron donor.*

**TABLE 4:** Ferric iron-reducing extreme acidophilic mixotrophic bacteria.

Organism	Fe ox.	S^0^ ox.	pH	Temp. [°C]	Conditions Fe red., ED	Growth	References
*Aciditerrimonas ferrireducens* ^T^	–	ND	3.0 2.0–4.5	50 35–58	Anaerobic: H_2_ or OC	+	Itoh et al., 2011
*Sulfobacillus acidophilus* ^T^	+	+	∼2.0	45–50	Anaerobic: S^0^ or OC	+	Johnson, 2010; Zhang et al., 2021
*Sulfobacillus thermosulfidooxidans* ^T^	+	+	1.7–2.4 1.5–5.5	50–55 20–60	Anaerobic: H_2_, K_2_S_4_O_6_ or OC	+	Bridge and Johnson, 1998; Hedrich and Johnson, 2013b; Zhang et al., 2021

*pH, temperature, sulfur oxidation and iron oxidation data correspond to the optima and range, which are described in the respective characterization publications. The growth column indicates whether dissimilatory iron reduction occurs under the given conditions. Temp, temperature; ox, oxidation; red, reduction; ED, electron donor; ND, no data; OC, organic carbon.*

**TABLE 5:** Ferric iron-reducing extreme acidophilic heterotrophic bacteria.

Organism	Fe ox.	S^0^ ox.	pH	Temp. [°C]	Conditions Fe red., ED	Growth	References
*Acidicaldus organivorans* ^T^	ND	+	2.5–3.0 1.75– > 3.0	50–55 40–65	Anaerobic: OC Micro-aerobic: OC	+ +	Johnson et al., 2006
*Acidiferrimicrobium australe* ^T^	+	–	3.0 1.7–4.5	30 20–39	Anaerobic: OC	+	González et al., 2020
*Acidimicrobium ferrooxidans* ^T^	+	–	2.0	45–50	Anaerobic: OC	+	Jones and Johnson, 2015
*Acidiphilium acidophilum* ^T^	–	+	2.0 2.0–4.5	30	Micro-aerobic: OC	+	Johnson and Bridge, 2002
*Acidiphilium* sp. SJH	ND	ND	2.0	28	Aerobic: OC Micro-aerobic: OC Anaerobic: OC	+ + ^−^	Johnson and Bridge, 2002
*Acidithrix ferrooxidans* ^T^	+	–	2.0–4.4	10–30	Anaerobic: OC Micro-aerobic: OC	+ +	Jones and Johnson, 2015
*Alicyclobacillus tolerans* ^T^	+	+	2.5–2.7 1.5–5.0	37–42 20–55	Anaerobic: OC	+	Karavaiko et al., 2005; Johnson, 2010
*Ferrimicrobium acidiphilum* ^T^	+	–	2.0 > 1.4	35 < 37	Anaerobic: OC	+	Johnson et al., 2009
*Ferrithrix thermotolerans* ^T^	+	–	1.8 > 1.6	43 < 50	Anaerobic: OC	+	Johnson et al., 2009
*Sulfobacillus benefaciens* ^T^	+	+	1.5 0.8–2.2	38.5 30–47	Anaerobic: OC	+	Johnson et al., 2008
*Sulfobacillus harzensis* ^T^	+	+	3.0 1.5–5.0	45 25–55	Anaerobic: OC	+	Zhang et al., 2021
*Sulfobacillus sibiricus* ^T^	+	+	2.0 1.1–2.6	55 17–60	Anaerobic: OC	+	Zhang et al., 2021
*Sulfobacillus thermotolerans* ^T^	+	+	2.0 1.2–2.4	40 20–60	Anaerobic: OC	+	Zhang et al., 2021

*pH, temperature, sulfur oxidation and iron oxidation data correspond to the optima and range, which are described in the respective characterization publications. The growth column indicates whether dissimilatory iron reduction occurs under the given conditions. Temp, temperature; ox, oxidation; red, reduction; ED, electron donor; ND, no data; OC, organic carbon.*

The investigation of the ferric iron reduction mechanisms in acidophiles is still ongoing and only few organisms, e.g., *Acidithiobacillus ferrooxidans*, were examined in detail. Over the years, various hypothesis were given, for instance an involvement of a nitrate reductase or tetrathionate hydrolase (Johnson and McGinness, 1991; Sugio et al., 2009), which could not be confirmed by later studies (Kucera et al., 2012, 2016b; Osorio et al., 2013). In addition to the biochemical process, it was crucial to determine whether anaerobic iron reduction is accompanied by cell growth or merely a mechanism for maintaining cellular functions. Initially, no growth was detectable for anaerobic iron-reducing *At. ferrooxidans* (Sugio et al., 1988), which was later contributed to an insufficient carbon dioxide supply (Das et al., 1992). Soon, anaerobic dissimilatory iron reduction was reported for different *At. ferrooxidans* strains (Pronk et al., 1991, 1992; Das et al., 1992). In this context, a doubling time of 24 h was determined for ferric iron-respiring *At. ferrooxidans*^T^ on elemental sulfur (Pronk et al., 1992) and the existence of two distinct electron transport systems for ferric iron reduction was suggested (Das et al., 1992). Additionally, the electron donor provided during pre-cultivation seemed to have a major impact on the ferric iron reduction ability of *At. ferrooxidans*. Cells grown on elemental sulfur for several generations were not able to reduce ferric iron when changed to anaerobic cultivation conditions, while the ones pre-cultivated with ferrous iron were (Kucera et al., 2012).

#### Uncovering Anaerobic Ferric Iron Reduction Mechanisms in Acidithiobacilli

**FIGURE 3:**
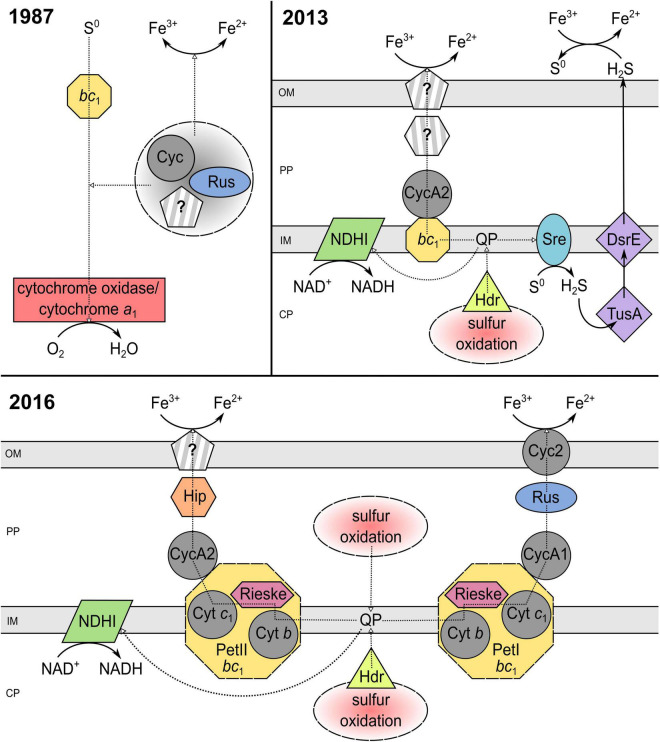
Elucidation of the iron reduction mechanism coupled to sulfur oxidation by *Acidithiobacillus ferrooxidans* from 1987 to 2016. The underlying models of Corbett and Ingledew (1987); Osorio et al. (2013), and Kucera et al. (2016a) were modified to a consistent style. PP, periplasm; CP, cytoplasm; OM, outer membrane; IM, inner membrane; Cyt, cytochrome; Cyc, cytochrome *c*; QP, quinol pool; Rus, rusticyanin; NDHI, NADH dehydrogenase complex; *bc*_1_, cytochrome *bc*_1_ complex (encoded by *petI/II*); Sre, sulfur reductase; DsrE/TusA, conserved hypothetical protein (likely sulfur transferase); Hdr, heterodisulfide reductase; Hip, high potential iron-sulfur protein; Rieske, Rieske iron-sulfur protein; ?, unidentified pathway component.

All to date described extreme acidophilic iron-oxidizing acidithiobacilli have been identified as anaerobic ferric iron reducers (Pronk et al., 1992; Hallberg et al., 2010; Hedrich and Johnson, 2013a; Falagán and Johnson, 2016; Norris et al., 2020). Accordingly, this genus offers great potential for research into the biochemical mechanisms of iron reduction, especially because *Acidithiobacillus ferrooxidans* has been studied in detail for its molecular processes as a flagship organism of bioleaching. Since the first investigation of anaerobic ferric iron reduction by *At. ferrooxidans* in the 1970s, a lot of effort has been put into the elucidation of the underlying enzymatic process. Sugio et al. (1985, 1987) purified and to some extent characterized a sulfur:ferric iron oxidoreductase from ferrous iron-grown *At. ferrooxidans* AP19-3. This periplasmic space enzyme catalyzed the oxidation of elemental sulfur to sulfite while reducing ferric to ferrous iron as one step of the sulfur metabolism and was therefore seen as an important part of the iron reduction chain (Sugio et al., 1985, 1987). In the same year, Corbett and Ingledew presented a model for the anaerobic iron respiration pathway of *At. ferrooxidans*^T^ (Figure 3) utilizing complexes and electron transporters of the aerobic iron oxidation chain (section “Metabolic Diversity of Extreme Acidophiles”). Thereby, the electron transfer from the donor elemental sulfur to the terminal acceptor iron may involve the cytochrome *bc*_1_ complex, cytochrome *c*, rusticyanin, cytochrome oxidase, cytochrome *a*_1_, and other transporters of the ferrous iron oxidation pathway located in the periplasmic space (Corbett and Ingledew, 1987). With this, the hypothesis arose that components of the iron oxidation chain could also be used in reverse for ferric iron respiration in acidophiles under anaerobic conditions, which was further investigated since then. Additional evidence was given by the observation, that *At. ferrooxidans* lacked the ability of anaerobic iron reduction after six consecutive aerobic cultivations on sulfur, which was contributed to the absence of some iron oxidation chain components in sulfur adapted cells (Kucera et al., 2012). Further, the investigation of anaerobic ferric iron respiration by *At. ferrooxidans* JCM 7811, later renamed as *At. ferriphilus* JCM 7811 (Kucera et al., 2020), with varying electron donors (hydrogen or elemental sulfur) resulted in the production of high amounts of a new c-type cytochrome (Ohmura et al., 2002). The purified cytochrome differed from other *At. ferrooxidans*-originating ones by an intermediate midpoint potential of about + 560 mV (pH 2.0) and was shown to act as an electron acceptor for ferric iron reduction (Ohmura et al., 2002). Consequently, the evidence was growing that cytochromes may play a major role in electron transfer during ferric iron reduction by sulfur-respiring acidophiles. The increased abundance of rusticyanin and cytochrome Cyc1 (both part of the ferrous iron oxidation pathway and encoded by the *rus* operon) in ferric iron-respiring *At. ferrooxidans* CCM 4253 further confirmed the contribution of *rus*-encoded, iron oxidation-related proteins in the iron reduction process (Kucera et al., 2012).

In 2013, Osorio et al. (2013) suggested two models for the anaerobic ferric iron reduction in *At. ferrooxidans*^T^ (Figure 3), based on microarray and proteomic examination during aerobic and anaerobic growth. One supported the aforementioned reverse utilization of the iron oxidation chain, by proposing the involvement of the *c*_4_-type cytochrome CycA2 and the *bc*_1_ complex. The second model introduced a new aspect, stating that an enzymatic disproportionation of sulfur during anaerobic sulfur oxidation may produce H_2_S which could mediate ferric iron reduction at low pH (Osorio et al., 2013). However, the ability of acidithiobacilli to grow anaerobically by coupling hydrogen oxidation (no H_2_S production) to iron reduction (Hedrich and Johnson, 2013b) suggested that ferric iron reduction mechanisms other than the indirect one must exist (Osorio et al., 2013). Still, the existence of an indirect iron reduction mechanism by H_2_S was not confirmed for *At. ferrooxidans* CCM 4253 due to no detection of H_2_S with lead acetate and a repressed expression of the sulfur reductase-encoding *sre* operon during anaerobic growth (Kucera et al., 2016a). Besides, multiple ferric iron reduction mechanisms were again indicated during comparative examination of strain CCM 4253 with and without ferric iron reduction ability (Kucera et al., 2015). Proteomic results showed 150 repressed spots of important proteins involved in the iron oxidation (e.g., rusticyanin, cytochrome Cyc2) and sulfur metabolism for cells lacking the iron reduction ability (Kucera et al., 2015). The induction of rusticyanin and Cyc2 in anaerobic iron-sulfur-respiring *At. ferrooxidans* implied that electrons may be collected by rusticyanin and transported to the outer-membrane cytochrome which could act as terminal ferric iron reductase (Kucera et al., 2015, 2016b). Moreover, transcriptional investigation of strain CCM 4253 coupling iron reduction to anaerobic sulfur oxidation revealed an overexpression of the *hip* gene encoding a high potential iron-sulfur protein (Hip) and thereby a new hypothetic iron reduction pathway in *At. ferrooxidans* (Kucera et al., 2016a). During aerobic growth, this iron-sulfur protein enables electron transfer between the quinone pool and the final oxidase, which reduces oxygen (Quatrini et al., 2006). Accordingly, the predicted, Hip-including anaerobic iron reduction mechanism suggested a transfer of electrons obtained through sulfur oxidation to take place from the quinone pool to the *bc*_1_ complex and is further transported by CycA2 and Hip to an unknown iron reductase (Kucera et al., 2016a; Figure 3). It has to be taken in consideration, that the pre-cultures used during the aforementioned anaerobic ferric iron reduction examinations were aerobically ferrous iron-grown, which invited Norris et al. (2018) to perform proteomic studies with anaerobically adapted cultures. Thereby, two further cytochromes, Cyc2B and Cyc1B, were detected and the allover results supported a participation of *petII*-encoded proteins (*bc*_1_ complex II and CycA2) in the anaerobic ferric iron reduction of sulfur-oxidizing *At. ferrooxidans*^T^ (Norris et al., 2018). This in turn reinforces the alternative ferric iron reduction mechanism using a high potential iron-sulfur protein as predicted by Kucera et al. (2016a).

**FIGURE 4:**
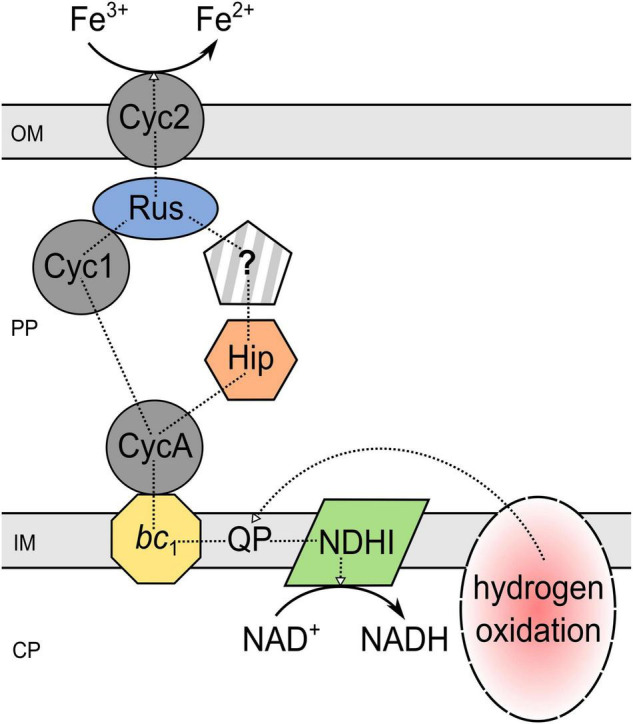
Proposed mechanism of iron reduction coupled to hydrogen oxidation by *Acidithiobacillus ferrooxidans*. The underlying scheme is given in Kucera et al. (2020). PP, periplasm; CP, cytoplasm; OM, outer membrane; IM, inner membrane; Cyc, cytochrome *c*; QP, quinol pool; Rus, rusticyanin; NDHI, NADH dehydrogenase complex; *bc*_1_, cytochrome *bc*_1_ complex (encoded by *petI/II*); Hip, high potential iron-sulfur protein; ?, unidentified pathway component.

To date, all the given insights into the biochemical iron reduction mechanism coupled to sulfur oxidation indicate the occurrence of at least two enzymatic, species-dependent iron reduction mechanisms in acidithiobacilli (Figure 3). The existence of an indirect process mediated by H_2_S needs further investigation, as the contradicting results of different strains do not imply a tendency. Nevertheless, there is a lot of evidence for both suggested electron transport chains from the inner to the outer membrane which may transfer electrons from the quinone pool to (i) the final reductase Cyc2 *via bc*_1_ complex I, CycA1, and rusticyanin; or (ii) a still unknown reductase by *bc*_1_ complex II, CycA2, and Hip (Corbett and Ingledew, 1987; Ohmura et al., 2002; Kucera et al., 2012, 2015, 2016a,2016b; Osorio et al., 2013; Norris et al., 2018). Analogous to ferric iron reduction during sulfur oxidation, the proposed mechanisms of anaerobic iron reduction coupled to hydrogen oxidation are only partially understood and are largely based on proteomic and transcriptomic analyses. In 2020, Kucera et al. (2020) introduced a model for anaerobic hydrogen metabolism in *At. ferrooxidans* CCM 4253 which suggests an electron transfer from hydrogen to iron *via* [NiFe] hydrogenase, ubiquinone pool, *bc*_1_ complex, cytochrome *c*_4_, Cyc1 or Hip, rusticyanin, and Cyc2. Consequently, the mechanisms that enable ferric iron reduction in an anaerobic environment may involve several similar enzymes and proteins which are also part of the ferrous iron oxidation pathway in acidophiles, regardless of the electron donor used (Figure 4). Therefore, it is likely, that organisms lacking these important components in their electron transport chain of iron oxidation (e.g., *rus*-encoded proteins), may not be able to enzymatically reduce ferric iron. However, there is still great research potential concerning these iron reduction processes. Only well-understood biochemical mechanisms can be optimally adapted to applications and supported by the required environmental conditions. Moreover, a detailed insight into the underlying microbial processes is key for the assessment of their large-scale implementation as biohydrometallurgical processes for reducing ferric iron minerals, such as goethite.

### Iron Reduction Under Micro-Aerobic Conditions

Besides iron-reducing processes under oxic or anoxic conditions, some acidophilic, heterotrophic microorganisms reduce ferric iron and/or mediate reductive dissolution of ferric iron minerals under micro-aerobic conditions. The type strains of *Acidibacter ferrireducens*, *Acidicapsa acidiphila*, and *Acidicapsa ferrireducens* catalyze the reductive dissolution of schwertmannite while metabolizing organic carbon sources (glucose, yeast extract) under oxygen-limitation (Falagán and Johnson, 2014; Falagán et al., 2017). Furthermore, heterotrophic, micro-aerobic *Acidicaldus organivorans*^T^**, *Acidobacterium capsulatum*^T^, *Acidocella aromatica*^T^, *Acidithrix ferrooxidans*^T^, and *Sulfolobus acidocaldarius*^T^ cultures showed iron-reducing properties (Johnson et al., 2006; Coupland and Johnson, 2008; Jones et al., 2013; Jones and Johnson, 2015; Masaki et al., 2018). In addition, several micro-aerophilic, acidophilic iron reducers perform this process under anaerobic conditions as well (Falagán and Johnson, 2014; Jones and Johnson, 2015), while others require low oxygen concentrations for this purpose (Falagán et al., 2017). In this context, discriminating between anaerobic, aerobic and micro-aerobic conditions is often difficult, as it is determined by the oxygen solubility under the given conditions. For an accurate classification, the oxygen content would have to be monitored during the entire experiment, which is not always feasible and involves more intricate experimental effort. Accordingly, some of the described aerobic iron reduction processes might actually occur under oxygen limitation, as has been discussed, for instance, for the thermophilic archaeon *Sulfolobus acidocaldarius* strain 79–13 (Brock and Gustafson, 1976). Therefore, a precise monitoring of the oxygen saturation in the culture medium is essential to obtain information on e.g., inhibition of iron reduction mechanisms by certain oxygen concentrations. This is particularly interesting to understand iron cycling at natural transition zones between oxic and anoxic habitats (Emerson et al., 2010).

#### Oxygen-Dependent Iron Reduction Processes of the Acidophilic (Chemo-) Organoheterotrophic Genus *Acidiphilium*

Comparative investigation on the impact of dissolved oxygen on ferric iron reduction by the extreme acidophile *Acidiphilium* spp. was conducted (Johnson and Bridge, 2002). Based on their different levels of aerobic iron reduction capacity (Johnson and McGinness, 1991), ferric iron reduction by *Acidiphilium acidophilum*^T^ and *Acidiphilium* sp. SJH was studied under various dissolved oxygen concentrations. Comparison of both, specific iron reduction rates and whole-cell protein profiles of these species, revealed oxygen-dependent differences within the genus *Acidiphilium* (Johnson and Bridge, 2002). *Acidiphilium* sp. SJH efficiently reduced ferric iron under aerobic and micro-aerobic conditions, demonstrated by nearly constant specific iron reduction rates between 40 and 80% dissolved oxygen. In contrast, effective iron reduction by *Acidiphilium acidophilum*^T^ was only observed under micro-aerobic conditions (20–40% dissolved oxygen). Both strains did not reduce iron in the absence of oxygen. The detection of three additional proteins in *Acidiphilium acidophilum*^T^ under micro-aerobic conditions indicated an oxygen-inducible iron reduction mechanism for this species. Since the dissolved oxygen content had no significant influence on the protein profile of *Acidiphilium* SJH, it was suggested to feature a constitutive iron reductase system. This tendency was also detected in further species of *Acidiphilium*, dividing the genus into two groups (Johnson and Bridge, 2002). However, *Acidiphilium cryptum* JF-5 reduced ferric iron independent of the given oxygen saturation even under anaerobic conditions at pH 3 (Küsel et al., 2002). Still, small amounts of oxygen are required to support growth of *Acidiphilium* species (Johnson and Bridge, 2002).

### Aerobic Iron Reduction at Extremely Low pH

In oxygen-rich environments, the detection of ferric iron reduction is hindered by the auto- and microbial oxidation of the generated ferrous iron. Consequently, the study of iron reduction processes under these conditions is challenging. The higher stability of ferrous iron at low pH values enables the monitoring of ferric iron reduction even in the presence of oxygen. Already in the 1970s, aerobic ferrous iron formation was reported for sulfur-oxidizing *Acidithiobacillus thiooxidans* and *Acidithiobacillus ferrooxidans* at low pH (Brock and Gustafson, 1976; Sand, 1989). Further, iron oxidation of *At. ferrooxidans* was non-detectable under these extremely acidic conditions. This led to the assumption that the iron oxidation chain of this species is inhibited below pH 1.3, preventing re-oxidation of the ferrous iron formed. A pH increase to 1.8 again enabled rapid ferrous iron oxidation (Sand, 1989). These results contradicted the until then prevailing assumption of exclusively anaerobic iron reduction by *At. ferrooxidans* (Brock and Gustafson, 1976). Also, strains belonging to the acidophilic, heterotrophic genus of *Acidiphilium*, were characterized as aerobic iron reducers, using glucose or glycerol as substrate (Johnson and McGinness, 1991). When comparing iron reduction rates, which are often used to obtain tendencies of organisms to reduce iron under certain conditions, it is important to respect what the numbers are referring to. In the case of *Acidiphilium cryptum* JF-5, the non-normalized ferric iron reduction rate of the anaerobic experiment (24.2 mmol L^–1^ day^–1^) was smaller than at oxygen saturation (46.6 mmol L^–1^ day^–1^), while normalizing the rate to cell density showed a lower iron reduction rate at oxygen saturation, which was attributed to better growth under these conditions (Küsel et al., 2002). Despite that, aerobic iron reduction was growth-related in further species, like *Acidithiobacillus thiooxidans*, *Acidiphilium cryptum*, and *Acidiphilium* sp. SJH (Brock and Gustafson, 1976; Johnson and McGinness, 1991). However, in *Sulfolobus acidocaldarius* and *At. ferrooxidans*, accumulation of ferrous iron was not accompanied by cell growth (Brock and Gustafson, 1976; Sand, 1989). Therefore, it remained unclear whether this was a microbial respiratory process or a non-enzymatic, chemical reduction mediated by metabolic intermediates accumulated in the culture liquor. Additionally, abiotic ferric iron reduction was shown for uninoculated flasks at thermophilic conditions (70°C). Still, the amount of iron reduced in inoculated batches was considerably higher (Brock and Gustafson, 1976).

#### Insights Into the Aerobic Ferric Iron Reduction Mechanisms in Acidithiobacilli

In recent years, aerobic ferric iron reduction ability was reported for various *Acidithiobacillus* species (Table 3) reducing soluble ferric iron and/or mediating the reductive dissolution of ferric iron ores (Marrero et al., 2015, 2017; Johnson et al., 2017; Smith and Johnson, 2018). The results obtained from these studies indicate species-dependent mechanisms for the “latent iron reduction” by acidithiobacilli (Johnson et al., 2021). Thereby, a non-growth-related mechanism was assumed for *At. thiooxidans* (Marrero et al., 2015), which contradicted the formerly proposed correlation between ferrous iron accumulation and cell growth in cultures of *At. thiooxidans* (Brock and Gustafson, 1976). Furthermore, cell-free supernatants (pH 1.0) of sulfur-respiring *At. caldus* and *At. ferridurans*, also showed aerobic iron reduction, indicating a cell-independent chemical reduction process (Johnson et al., 2017). However, in the case of *At. thiooxidans*, washed cells were also capable of reducing iron within a short time under aerobic conditions (150–200 mg/L in 6.5 h) regardless of the pH (0.9, 2.5, 3.5) (Marrero et al., 2015). Initially, the aerobic iron reduction observed in *Acidithiobacillus* (especially *At. thiooxidans*) cultures was exclusively assigned to the reduction of ferric iron by sulfur intermediates formed during sulfur respiration under aerobic conditions (Marrero et al., 2015, 2017). However, Johnson et al. (2017, 2021) provided evidence, that aerobic ferric iron reduction mediated by acidithiobacilli is not only attributed to sulfur oxidation, as hydrogen-grown *At. caldus* and tetrathionate- or hydrogen-metabolizing *At. thiooxidans* reduced ferric iron under these conditions as well. Recently, indications were given that an initial hydrophilization of sulfur by acidithiobacilli may enable chemical, ferric iron reduction as tests with bio-activated, heat-sterilized sulfur allowed aerobic iron reduction at 50°C and pH 0.9–1.0 as well (Johnson et al., 2021). These finding may also explain the pH-independent iron reduction of washed *At. thiooxidans* cells by cell-surface-associated activated sulfur particles. Accordingly, further studies are required to gain more insight into the processes that occur during aerobic iron reduction at low pH. Moreover, the aforementioned aerobic reduction of ferric iron by heterotrophic *Acidiphilum* species (Johnson and McGinness, 1991) implies that there are several mechanisms enabling ferrous iron formation in the presence of oxygen. Whether these mechanisms are enzymatic, non-enzymatic, or a combination of both should be further investigated. In this context, the detection and identification of intermediates of the sulfur metabolism is a major drawback due to their low stability and rapid degradation (Rohwerder, 2002).

## Application and Environmental Aspects

Reductive dissolution by acidophiles was shown for several ferric iron-containing minerals, as ferric hydroxide [Fe(OH)_3_], goethite (α-FeOOH), akageneite (β-FeOOH), magnetite (Fe_3_O_4_), potassium jarosite [KFe_2_(SO_4_)_2_(OH)_6_], and natrojarosite [NaFe_2_(SO_4_)_2_(OH)_6_] (Bridge and Johnson, 1998, 2000). These findings opened new possibilities for biohydrometallurgical processes where oxidative bioleaching is not effective or more sustainable methods should be established. Nevertheless, as other metal recovery methods, anaerobic (AnRD) and aerobic reductive dissolution (AeRD) require special operating conditions which have to be considered especially regarding operational costs. The maintenance of an anaerobic process requires a closed system and exclusion of oxygen, e.g., by supply of nitrogen. Further, AnRD is an acid-consuming reaction involving the addition of acid (Marrero et al., 2015), while AeRD can operate as net acid-producing process (Johnson et al., 2021). On the downside, the extremely low pH during AeRD is only tolerated by few microorganisms, which minimizes the number of suitable acidophiles. Moreover, the exclusion of aerobic iron oxidizers from AeRD applications may require an anaerobic phase integrated into the process to avoid interference with iron-oxidizing leptospirilli (Marrero et al., 2015). Still, reductive bioleaching is a promising method to exploit even low-grade ferric iron minerals and to recycle process wastes of traditional hydrometallurgical methods, e.g., laterite tailings (Marrero et al., 2015).

### Biohydrometallurgical Application of Microbial Iron Reduction

The success of biotechnological applications in the metallurgical sector is evident from copper production *via* bioleaching in e.g., Chile and elsewhere in the world (Gentina and Acevedo, 2016). Despite the fact that biohydrometallurgical processes are nowadays well established, commercial applications are still limited to sulfidic minerals (Johnson, 2014). In the case of oxidative metal dissolution by acidophiles, the process is enabled by the sulfuric acid produced during sulfur oxidation by the microorganisms and/or the ferric iron generated by microbial iron oxidation of the mineral (Johnson et al., 2013). The mechanism underlying reductive dissolution of ferric iron minerals is also caused, to some extent, by acidolysis (Marrero et al., 2015). Further, an equilibrium shift between iron in the solid phase and in solution created by microbial ferric iron reduction accelerates mineral dissolution (Bridge and Johnson, 1998). Although further research is required to understand the exact mechanisms involved, reductive bioleaching is already enabling new, economic recovery methods for different ferric iron-associated metals in laboratory scale.

#### Processing Ferric Iron Ores for Base Metal Recovery

Over the last decade, a lot of research has been conducted on the application of reductive microbial dissolution processes focusing on the extraction of metals from limonitic laterites (Table 6). Approximately 70% of world’s land-based nickel resources are present in the form of laterite (Dalvi et al., 2004). Laterites are commercially processed by reagent- and energy-demanding pyrometallurgical (smelting) or hydrometallurgical (CARON process or high pressure acid leaching) processes (Dalvi et al., 2004). Nickel laterites consist of two zones, the upper limonite and the lower saprolite zone, of which the limonite one contains base metals associated with ferric iron in the form of modified goethite (Johnson et al., 2013). Bioleaching of limonite by acidithiobacilli is achieved through microbial acid generation, lowering the redox potential under anaerobic conditions, iron reduction under aerobic conditions and activating/“wetting” of naturally hydrophobic sulfur (Johnson et al., 2021). Advantages compared to conventional pyro- and hydrometallurgical methods are cost reduction by using inorganic energy sources (e.g., sulfur) and an ambient operating temperature (Johnson et al., 2013). The extremely low pH during AeRD and AnRD enhances the acidic attack during mineral dissolution and reduces metal precipitation, thereby simplifying downstream processing which is a major advantage compared to neutrophilic iron reduction approaches (Johnson et al., 2013).

**TABLE 6:** Overview of reductive mineral dissolution using extreme acidophiles.

Mineral	Origin	Microorganisms	Method	Yield	References
Nickel laterite	Western Australia	*At. ferrooxidans* ^T^	AnRD	> 80% Ni	Hallberg et al., 2011a
Nickel laterite	Western Australia	*At. ferrooxidans* ^T^	AeRD	∼10% Ni	Hallberg et al., 2011a
Limonitic nickel laterite	Australia	*At. ferrooxidans* ^T^	AnRD	46–82% Ni 64–90% Co 75–116% Mn	Johnson et al., 2013
Copper laterite	Carajás Belt, Pará State, Brazil	*At. ferrooxidans* ^T^	AnRD	≤ 78% Cu	Nancucheo et al., 2014
Laterite tailings	Moa, Cuba; from CARON process	*At. thiooxidans* ^T^	AeRD	53% Ni 46% Co	Marrero et al., 2015
Laterite tailings	Moa, Cuba; from CARON process	*At. thiooxidans*^T^ and *At. ferrooxidans*^T^	AeRD	56% Ni 60% Co	Marrero et al., 2015
Laterite tailings	Moa, Cuba; from CARON process	*At. thiooxidans*^T^ and *At. ferrooxidans*^T^	Ae-AnRD	53% Ni 58% Co	Marrero et al., 2015
Laterite tailings	Moa, Cuba; from CARON process	*At. ferrooxidans* ^T^	AnRD	58% Ni 56% Co	Marrero et al., 2015
Nickel laterite overburden	Punta Gorda, Moa, Cuba	*At. thiooxidans* ^T^	AeRD	16% Ni 85% Co 74% Mn	Marrero et al., 2017
Nickel laterite overburden	Punta Gorda, Moa, Cuba	*At. ferrooxidans* ^T^	AnRD	16% Ni 78% Co 86% Mn	Marrero et al., 2017
Cobalt-bearing limonitic laterite ore	Shevchenko, Kazakhstan	Consortium mesophilic and thermotolerant bacteria^a^	AnRD	50–70% Ni 89–99% Co	Smith et al., 2017
Cobalt-bearing limonitic laterite ore	Acoje mine, Philippines	Consortium mesophilic and thermotolerant bacteria^a^	AnRD	40% Ni 90% Co	Smith et al., 2017
Cobalt-bearing limonitic materials	Kastoria, Agios loannis and Evia mine, Greece	Consortium mesophilic bacteria^b^	AnRD	37–73% Ni 40–50% Co 15–52% Mn	Santos et al., 2020
Limonite ore	Shevchenko, Kazakhstan	Consortium mesophilic bacteria^c^	AnRD	∼55% Ni ∼90% Co ∼95% Mn	Johnson et al., 2021
Limonite ore	Shevchenko, Kazakhstan	Consortium mesophilic bacteria^c^	AeRD	∼25% Ni ∼20% Co ∼40% Mn	Johnson et al., 2021
Limonite ore	Shevchenko, Kazakhstan	*At. caldus* ^T^	AeRD	∼100% Ni ∼95% Co ∼90% Mn	Johnson et al., 2021

*^a^At. ferrooxidans^T^, At. ferriphilus^T^, At. ferridurans^T^, “Acidibacillus sulfuroxidans”^T^, Sulfobacillus thermosulfidooxidans^T^, and Sulfobacillus acidophilus BOR1.*

*^b^At. ferrooxidans^T^, At. ferrooxidans CF3, At. ferriphilus^T^, At. ferridurans^T^, and Sulfobacillus thermosulfidooxidans^T^.*

*^c^At. ferrooxidans^T^, At. ferriphilus^T^, At. ferridurans^T^, Sulfobacillus acidophilus^T^, and Sulfobacillus thermosulfidooxidans^T^. AnRD, anaerobic reductive dissolution; AeRD, aerobic reductive dissolution; Ae-AnRD, aerobic reductive dissolution with an integrated anaerobic phase.*

According to the diverse composition of laterites, different base metals have already been the target of reductive bioleaching, such as nickel (Hallberg et al., 2011a), copper (Nancucheo et al., 2014), and cobalt (Smith et al., 2017), reaching up to 100% yield. It also became apparent, that the geographical origin of the ore and its mineralogical composition had a major impact on their biohydrometallurgical application (Santos et al., 2020). Initially, the applicability of reductive bioleaching was examined by using pure cultures of *Acidithiobacillus ferrooxidans* operating under anoxic conditions (Hallberg et al., 2011a; Johnson et al., 2013; Nancucheo et al., 2014). Afterward, other acidophiles and consortia as well as different aeration setups (AeRD, Ae-AnRD and AnRD) were investigated for this reductive extraction approach (Marrero et al., 2015; Santos et al., 2020; Johnson et al., 2021). In some processes, also thermotolerant strains were used (Smith et al., 2017; Johnson et al., 2021) which enable higher operation temperatures and thereby enhanced reaction kinetics. However, the use of consortia also showed that the diversity of the microbial community decreases during the reductive bioleaching process, which could sometimes be due to a lack of adaptation of the acidophiles to the mineral, resulting in the predominance of a few strains (Smith et al., 2017; Santos et al., 2020). Monitoring of cell numbers by qPCR also showed a constant number of active bacteria during the AeRD process and a decrease after 3 days in the case of AnRD and Ae-AnRD (Marrero et al., 2015). Moreover, comparison of reductive limonite dissolution by a mesophilic consortium of *At. ferrooxidans*^T^, *At. ferriphilus*^T^, *At. ferridurans*^T^, *Sulfobacillus acidophilus*^T^, and *Sulfobacillus thermosulfidooxidans*^T^ showed higher base metal yields in the anaerobic bioreactor, while the aerobic bioreactor required no further acid supply after 9 days (Johnson et al., 2021). Smith et al. (2017) observed the same tendency for cobalt extraction from limonite, which was more efficient using the anaerobic reductive leaching approach and seemed to correlate with manganese dissolution. In contrast, AeRD experiments with pure cultures of non-iron-oxidizing acidithiobacilli (*At. thiooxidans* or *At. caldus*) resulted in higher metal yields compared to AnRD approaches with the same limonite (Marrero et al., 2017; Johnson et al., 2021). Marrero et al. (2017) provided a direct comparison of AnRD and AeRD of nickel laterite overburden by *At. ferrooxidans* or *At. thiooxidans*, respectively, showing approximately the same yields. Accordingly, in this case AeRD was selected as the favorable method since it does not require anoxic conditions, it enables better extraction kinetics, and the extremely low pH reduces iron precipitation (Marrero et al., 2017). The success of bioprocessing of limonitic ores depends on the mineralogy. If large amounts of acid-unstable ferrous iron minerals are present, manganese-associated cobalt is readily solubilized by microbial acid generation under aerobic conditions, whereas ferric iron reduction by acidophiles promotes solubilization of ferric iron minerals and nickel (Johnson et al., 2021). In addition, microbial ferrous iron generation accelerates the reductive dissolution on Mn (IV) minerals (Santos et al., 2020; Johnson and Pakostova, 2021).

All this indicates that, as in any biotechnological process, a large number of parameters need to be optimized to obtain the best possible results. These are highly influenced by the mineral composition, the bioleaching method and the microorganisms involved. Accordingly, pre-investigations concerning the characteristics of the applied acidophiles and ores are crucial. Furthermore, abiotic effects during reductive bioleaching must be considered. In the context of abiotic effects caused by chemical leaching *via* acid dissolution at extremely low pH values used for AnRD and AeRD, it was shown that bacterial catalyzed approaches with iron reducers allow more effective metal dissolution than acidolysis alone (Nancucheo et al., 2014). Reductive bioleaching was also up to 6 times more effective than acid leaching in processing cobalt-containing limonite from Kazakhstan or the Philippines under aerobic conditions (Smith et al., 2017). Further benefits of reductive bioleaching approaches were shown by an initial anaerobic phase during leaching of polymetallic sulfides which had to some extend positive effects on the base metal recovery and enhanced the iron removal noticeable at both 45 and 70°C (Norris et al., 2015).

Additional to the bioleaching of base metals from laterites, iron reduction by acidophiles offers far more application potential. The bio-reductive processes would be especially suitable to recycle scrap electronics or bioremediate metal-contaminated soil (Johnson et al., 2013). Another approach aimed at the pretreatment of laterite-associated monazite, a rare earth elements-containing phosphate mineral, by ferric iron reduction to improve the monazite exposure for further acid dissolution (Nancucheo et al., 2019). However, due to the innovation of this method, further research is required before it is ready for application.

#### Introducing a Reductive Bioleaching Circuit: The Ferredox Concept

In 2011, a generic concept for ferric iron mineral processing, Ferredox, was presented for tropical limonitic laterite (du Plessis et al., 2011). In assembly, the model has not yet been applied in a full-scale operation, but it has been proposed as a promising sustainable metal recovery method. The modular process relies on the findings that anaerobic reductive dissolution of goethite can take place under ambient conditions utilizing bacterial catalysis (Hallberg et al., 2011a). This new perspective creates a biohydrometallurgical method, which might allow iron oxide treatment with reduced material and processing costs. Overall, the process consists of four modularized main stages: reductive bioleaching, metal recovery, iron oxidation/precipitation, and reductive acid generation. During the initial reductive bioleaching, iron-oxidizing/reducing acidithiobacilli couple the oxidation of elemental sulfur to the reduction of ferric iron present in goethite and other minerals, solubilizing target metals. The resulting pregnant leaching solution is subsequently processed to extract the focused base metals. After a two staged iron oxidation process, the formed iron precipitates are utilized to generate the sulfuric acid consumed during the initial reductive dissolution of goethite [S^0^ + 6FeO(OH) + 10H^+^ → SO_4_^2–^ + 6Fe^2+^ + 8H_2_O]. In turn, this process will make use of the metabolism of *At. ferrooxidans* once again by anaerobic oxidation of elemental sulfur generating acid (S^0^ + 6Fe^3+^ + 4H_2_O → SO_4_^2–^ + 6Fe^2+^ + 8H^+^). Advantages of the given Ferredox concept are biomass recycling and enhancement due to the use of *At. ferrooxidans* in multiple process steps and increased sustainability due to biologically catalyzed reactions and operation at ambient conditions (du Plessis et al., 2011; Johnson and du Plessis, 2015).

### Environmental Impact of Acidophilic Iron Reducers

In an ancient, oxygen-free atmosphere, the usage of ferric iron as electron acceptor may have had a major role and impact on live (Walker, 1987; Slobodkin, 2005). Currently, oxido-reduction of iron has been demonstrated for both pure and mixed cultures of acidophiles (Johnson et al., 1993). It is now known that microbial iron cycling effects inorganic and organic contaminants, carbon cycling, and nutrient mobilization in the environment (Kappler and Straub, 2005). For instance, iron-reducing acidophiles were shown to eliminate the toxicity of some oxy-anions through ferrous iron generation, e.g., the more toxic Cr(VI) (hydrogen chromate, HCrO_4_^–^) is reduced to the less harmful Cr(III) by ferrous iron (Johnson et al., 2017).

Again, the dissolution of iron oxides releases metal cations absorbed to the mineral, which may have a negative impact on the biosphere (Kappler and Straub, 2005). Nevertheless, ferrous iron, the product of ferric iron reduction, can in turn act as a strong reductant for organic pollutants, e.g., nitroaromatics (Hofstetter et al., 1999).

The fact that acidophilic iron reducers feature autotrophic as well as heterotrophic metabolisms ensures their function as both, producers and degraders in ecosystems (Kashefi et al., 2004). The resulting complete mineralization of organic matter prevents accumulation of high amounts of organic substances which could have a toxic effect, e.g., small organic acids, on autotrophic acidophilic microorganisms. In acid streamers, dead organic matter and generated iron precipitates settle down to anoxic, organotrophic acidophiles-inhabitated zones, which were shown to feature more ferrous than ferric iron due to the occurring oxidation of organic substances coupled to iron reduction (Johnson et al., 1993, 2012). Thus, acidophiles actively contribute to the carbon and iron cycle. In the river Rio Tinto, iron transformation was shown to be mainly enabled by the interaction of the autotrophic iron oxidizers *Leptospirillum ferrooxidans* and *At. ferrooxidans* in aerobic areas and iron reduction by *At. ferrooxidans* (autotrophic) and *Acidiphilium* species (heterotrophic) in anaerobic zones (González-Toril et al., 2003). Investigations of the microbial diversity in sediments of an acidic lignite mine lake (Lusatia, Germany) revealed further iron reducers and cycling in these environments. Isolates closest related to species of *Acidiphilium*, *Acidocella*, *Ferrimicrobium*, *Acidithiobacillus*, *Alicyclobacillus*, and *Acidobacteriaceae* reduced iron in this habitat, with some of them being found throughout all zones of the sediment (Lu et al., 2010). Likewise, previous investigations of the same coal mine lake emphasized this pH gradient-dependent heterogeneity of ferric iron-reducing acidophiles in the sediment (Blöthe et al., 2008). In addition, two of the isolated *Alicyclobacillus* relatives showed iron cycling capacity in the laboratory by dissolving schwertmannite and re-oxidizing the ferrous iron by forming schwertmannite precipitates again (Lu et al., 2010). Still, the capacity of iron cycling by pure cultures was previously described for other acidophilic isolates (Johnson et al., 1993). Nevertheless, in many acidic ecosystems, a diverse community of phyla is responsible for the conversion of iron and other elements. Depending on the conditions, a closed iron cycle can be independently realized by individual species. According to the manifold influences of acidophilic iron reducers on different ecosystems and material cycles, it is important to study this group of microorganisms and thus to achieve a better awareness of human interventions, especially with regard to anthropogenically induced acidic and metal-rich habitats.

## Conclusion and Perspectives

Although iron reduction processes mediated by acidophiles have been known for nearly 50 years, it is only in the last decade that much interest has developed in their application. This especially included the utilization of well-studied ferric iron reducers of the genus *Acidithiobacillus*. Nevertheless, far more iron-reducing acidophiles have been identified including over 20 different genera. This is justified by the growing impact of this metabolic trait due to the recently known suitability for reductive bioleaching of ferric iron minerals. Accordingly, the characterization of novel acidophilic microorganisms now usually includes testing for iron reduction, which was not necessarily the case in the past. Still, few of the newly discovered iron reducers are investigated in detail. This lack of knowledge offers a great demand for further research particularly with regards to thermophilic conditions which could enhance the leaching kinetics. Moreover, the different mechanisms of ferric iron reduction in acidophiles (anaerobic, micro-aerobic or aerobic) allow diverse operation setups for base metal processing from laterites and a correspondingly higher freedom to customize the metal extraction method according to the mineral composition. Recently, the high potential to use acidophile-promoted reductive dissolution was demonstrated by exceeding base metal extraction from ferric minerals and overburden. Approaches were applied to recycle low-grade wastes from traditional laterite processing by reductive bioleaching. Additionally, first approaches to pretreat rare earth-bearing minerals associated with laterites showed promising results. Besides, ferric iron reduction by acidophiles might further be suitable for bioremediation and scrap electronics recycling. All the benefits of such an innovative application profit from precise knowledge of the underlying microbiological processes and mineral composition. Both call for suitable devices and expertise in microbiological and mineralogical fields which may in some cases rely on an interdisciplinary cooperation. Further adjustments of this biohydrometallurgical approach require a sufficient understanding of the biochemical iron reduction mechanism, especially concerning acidophiles apart from acidithiobacilli. As *Sulfobacillus thermosulfidooxidans* was shown to be the predominant microorganism (beside *Acidithiobacillus ferrooxidans*) in reductive bioleaching experiments with moderate consortia, this acidophile seems to be an outstanding candidate for detailed investigations of its iron reduction mechanism. The biochemical information obtained for *At. ferrooxidans* offered new knowledge and interesting insights, by showing a reverse usage of iron oxidation chain components. Accordingly, a comprehensive insight into the iron reduction mechanisms of other acidophiles, especially those with iron oxidation pathways other than *At. ferrooxidans*, would be of great interest. The research field of reductive bioleaching would benefit from further studies on additional acidophilic genera and even archaea.

## Author Contributions

LM: writing manuscript, preparing figures, and tables. SH: conceptual discussions, revising, and editing manuscript. Both authors contributed to the article and approved the submitted version.

## Conflict of Interest

The authors declare that the research was conducted in the absence of any commercial or financial relationships that could be construed as a potential conflict of interest.

## Publisher’s Note

All claims expressed in this article are solely those of the authors and do not necessarily represent those of their affiliated organizations, or those of the publisher, the editors and the reviewers. Any product that may be evaluated in this article, or claim that may be made by its manufacturer, is not guaranteed or endorsed by the publisher.
